# Dual RNA-Seq characterization of host and pathogen gene expression in liver cells infected with Crimean-Congo Hemorrhagic Fever Virus

**DOI:** 10.1371/journal.pntd.0008105

**Published:** 2020-04-06

**Authors:** Robert A. Kozak, Russell S. Fraser, Mia J. Biondi, Anna Majer, Sarah J. Medina, Bryan D. Griffin, Darwyn Kobasa, Patrick J. Stapleton, Chantel Urfano, Giorgi Babuadze, Kym Antonation, Lisa Fernando, Stephanie Booth, Brandon N. Lillie, Gary P. Kobinger

**Affiliations:** 1 Department of Laboratory Medicine & Molecular Diagnostics, Division of Microbiology, Sunnybrook Health Sciences Centre, Toronto, Ontario, Canada; 2 Department of Pathology and Microbiology, Atlantic Veterinary College, University of Prince Edward Island, Charlottetown, Prince Edward Island, Canada; 3 Arthur Labatt Family School of Nursing, Western University, London, Ontario, Canada; 4 Toronto Centre for Liver Disease, Toronto General Hospital, University Health Network, Toronto, Ontario, Canada; 5 National Microbiology Laboratory, Public Health Agency of Canada, Winnipeg, Manitoba, Canada; 6 Department of Medical Microbiology and Infectious Diseases, University of Manitoba, Winnipeg, Manitoba, Canada; 7 Department of Laboratory Medicine and Pathobiology, University of Toronto, Toronto, Ontario, Canada; 8 Infectious Diseases Research Centre, Université Laval, Quebec City, Quebec, Canada; 9 Department of Pathobiology, Ontario Veterinary College, University of Guelph, Guelph, Ontario, Canada; Northeastern University, UNITED STATES

## Abstract

Crimean-Congo hemorrhagic fever virus (CCHFV) is a tick-borne virus that can cause a hemorrhagic fever in humans, with a case fatality rate of up to 40%. Cases of CCHFV have been reported in Africa, Asia, and southern Europe; and recently, due to the expanding range of its vector, autochthonous cases have been reported in Spain. Although it was discovered over 70 years ago, our understanding of the pathogenesis of this virus remains limited.

We used RNA-Seq in two human liver cell lines (HepG2 and Huh7) infected with CCHFV (strain IbAr10200), to examine kinetic changes in host expression and viral replication simultaneously at 1 and 3 days post infection. Through this, numerous host pathways were identified that were modulated by the virus including: antiviral response and endothelial cell leakage. Notably, the genes encoding DDX60, a cytosolic component of the RIG-I signalling pathway and OAS2 were both shown to be dysregulated. Interestingly, *PTPRR* was induced in Huh7 cells but not HepG2 cells. This has been associated with the TLR9 signalling cascade, and polymorphisms in *TLR9* have been associated with poor outcomes in patients. Additionally, we performed whole-genome sequencing on CCHFV to assess viral diversity over time, and its relationship to the host response. As a result, we have demonstrated that through next-generation mRNA deep-sequencing it is possible to not only examine mRNA gene expression, but also to examine viral quasispecies and typing of the infecting strain. This demonstrates a proof-of-principle that CCHFV specimens can be analyzed to identify both the virus and host biomarkers that may have implications for prognosis.

## Author summary

Crimean-Congo hemorrhagic fever virus (CCHFV) is an understudied tick-borne virus that can cause a wide spectrum of disease, ranging from moderate to severe, and can be fatal. Cases have been reported in Asia, Africa and Europe, but the range of the vector continues to expand. Currently, our understanding of the host innate immune response to the virus remains limited. Our aim was to use RNA-seq, a type of next generation sequencing technology, to characterize the host immune response in liver cells as well as sequence the genome of the virus. Results identified numerous genes and pathways that were altered and served as proof of principle that the viral identification and evolution could be investigated from the same sample simultaneously. This study highlights the potential for this technique for both characterization of the virus as well as identification of host biomarkers that could be potential predictors of patient outcome. Additionally it may provide important information about pathogenesis prior to performing animal infection studies.

## Introduction

Crimean-Congo hemorrhagic fever virus (CCHFV) is a tick-borne virus that can cause a hemorrhagic fever in humans, with a case fatality rate of up to 40% [1]. Cases of CCHFV have been endemic in Africa, Asia, and South-Eastern Europe for over 70 years, and, during the recent years, autochthonous cases have also been reported in Spain due to the expanding range of its tick vector species [2]. The virus has several animal hosts, including agriculturally important animals such as cattle and goats. Transmission to humans occurs as a result of bites from infected ticks or via exposure to body fluids from viremic animals or humans [3]. Therefore, nosocomial transmission to healthcare workers is an important concern [4, 5].

Although CCHFV was discovered over seven decades ago, our understanding of the pathogenesis remains limited. A hallmark of CCHFV infection is the increase in vascular permeability, likely due to impaired endothelial cell function [6], that results in the characteristic hemorrhaging observed in clinical cases. However, CCHFV has been shown *in vitro* to infect numerous cell types, including mononuclear cells, epithelial cells, and hepatocytes [6–8]. Several studies suggest that the liver is an important target organ for the virus. For example, the virus has been shown to replicate to higher titers in Huh7 cells, compared to other non-hepatocytes lines [6–8]. Additionally, the highest viral titers were observed in the livers of STAT-1 knockout mice infected with CCHFV, and in the recently published cynomolgus macaque model, hepatic necrosis was noted [9, 10]. Furthermore, clinical findings also support the role of the liver in disease, as it is an early target organ for CCHFV [11], and its involvement following infection is associated the elevation of AST and ALT correlating with poor prognosis [11–13]. This suggests that understanding the host response in liver cells could provide insight into CCHFV pathogenesis.

Similar to other viral pathogens, CCHFV encounters a changing intracellular host environment during the course of infection. Dual RNA-Seq is a technique that has demonstrated that it is possible to study both pathogen evolution and host response simultaneously [14], allowing for examination of host response and, in the case of RNA virus, viral adaptation over the course of infection. The approach has the advantage over microarray studies in that it may identify RNA species that would not present on a microarray chip [15]. Therefore, it may be possible to characterize the host mRNAs that undergo a change in expression, leading to the identification of potential biomarkers [16, 17]. Simultaneously, sequencing the virus over time may also lead to the identification of novel variants, which could be of particular clinical importance in outbreak settings. Additionally, analysis of the viral sequences also permits genotyping that could assist with epidemiological studies.

In this study we used RNA-Seq in two human liver cell lines infected with CCHFV, to examine kinetic changes in host expression. Simultaneously, we whole-genome sequenced CCHFV to assess viral diversity over time, and its relationship to the host response. As a result, we have demonstrated that through next-generation mRNA deep-sequencing it is possible to not only examine mRNA gene expression, but also to examine viral evolution. We have also identified key pathways, which are upregulated or downregulated in liver cell lines, which may be involved in endothelial cell leakage in the context of CCHFV.

## Methods

### Virus and cells

HepG2 and Huh7 are well-differentiated hepatocarcinoma cell lines derived from a pediatric hepatoblastoma and an adult hepatocarcinoma biopsy, respectively [18–21].Cells were generously provided by Dr. Jordan Feld, Toronto Centre for Liver Disease, Toronto General Hospital and Dr. Michael Carpenter, Viral Diseases Division, National Microbiology Laboratory; and were maintained in Dulbecco’s modified eagle media, supplemented with 10% fetal bovine serum, 2mM L-glutamine, and penicillin (100 IU/ml)/streptomycin (100μg/ml). All infection studies used Crimean Congo Hemorrhagic Fever virus (CCHFV) IbAr 10200 (accession numbers AY389508, AF467768, and CHU88410). This virus had been passaged 10 times in suckling mice and 3 times in SW-13 cells. Complete details are described by Bente et al [22]. To assess viral replication and gene expression, cells were seeded into 6-well plates and infected at a MOI of 0.1 for 1 h at 37°C. Following this, the inoculum containing the virus was removed and cells were washed with 1X PBS and fresh media was added. At predetermined time points the supernatant was removed and viral titer determined by RT-PCR as described previously [22]. For gene expression experiments mock infection controls were performed in parallel and harvested in the same manner at the same time points as the infected cells (n = 3). To assess viral replication, cells were infected at a multiplicity of infection (MOI) of 0.1 PFU for 1 h at 37°C. Following this, the inoculum containing the virus was removed, cells were washed with phosphate-buffered saline (PBS), and fresh media was added. At predetermined time points the supernatant was removed and viral titer determined by RT-PCR using the methods outlined by Pang et al [23]. Primers and probes used were as follows: F(726–747): GCCGTTCAGGAATAGCACTTGT; R(869–889):TGTTATCATGCTGTCGGCRCT;P(750–777):HEX- CAACAGGCCTTGCYAAGCTYGCAGAGAC-BHQ1. All experiments were performed in the Biosafety Level 4 containment laboratory of the Canadian Science Centre for Human and Animal Health (CSCHAH) in Winnipeg, Canada.

### mRNA sequencing

For host transcriptome analysis, infected and uninfected cells were harvested at 1- and 3-days post-infection. Total RNA was isolated from cells using the Qiagen RNeasy kit according to manufacturer’s instructions. Next generation sequencing (NGS) libraries were prepared using TrueSeq Stranded mRNA kit (Illumina) following manufacturer’s recommendations for low sample input. A total of 500 ng RNA was used as input and libraries were amplified using 15 PCR cycles. Libraries were size selected via the Blue Pippin system (Sage Science) to isolate ~ 260 bp fragments. Libraries were verified on a High Sensitivity DNA chip using the 2100 Bioanalyzer (Agilent). Sequencing was performed by GenomeQuebec on a HiSeq4000 Illumina system by multiplexing 12 samples per flow cell and performing 100 bp paired-end reads.

### mRNA expression analysis

Raw reads were trimmed using Trimmomatic [24] requiring a minimum read length of 30 bp, a minimum Phred score of 20 over a 5 bp sliding window, and trimmed trailing base pairs with a Phred score of 19 or less. Raw reads were aligned to the GRCh38 release of the human genome [25] using HISAT2 v.2.1. [26] with the ‘–dta’ option enabled for downstream transcriptome assembly with StringTie; remaining options were left at their default settings. Quality of the alignment was evaluated using the RSeQC v2.6.4 package [27] ensuring uniform gene body coverage and adequate known junctional coverage. StringTie v.1.3.3b was used to assemble the transcriptome [26] in a two-step process. Following primary assembly for each sample, all assemblies were merged into an experiment-level reference assembly and the abundances of all transcripts were then re-estimated based on the experiment-level reference. Aligned sequencing data and GTF files used in the analysis of CCFHV-infected cell lines are publicly available in the NCBI GEO Repository under accession number GSE133086.

Differential expression analysis was performed using the DESeq2 v1.18.1 package [28] for R [29]. The data was reformatted for analysis in DESeq2 using the “prepDE.py” script available at (https://ccb.jhu.edu/software/stringtie/index.shtml?t=manual). Differential expression between uninfected and infected cells was evaluated at each time point for each cell line. In order to account for variation in read depth and library composition, an internal normalization using a scaling factor derived from the median-of-ratios methods was performed using DESeq2 [30]. DESeq2 was then used to fit each gene to a generalized linear model following a negative binomial distribution, and significance was assessed using a Wald test. A fold-change of 1.5 or greater was considered significant at a p-value of 0.05 or less after correction for multiple testing using the Benjamini-Hochberg procedure. Genes with 0 or 1 transcripts across replicates were removed from the analysis. Bioinformatic prediction of mRNA interactions and pathway analyses was performed using Reactome database (www.reactome.org). A core pathway analysis on this list was performed to determine the functional or biological relevance of these genes, and their relationships. Data is from three biological replicates.

### Whole-genome sequencing of CCHFV

**Whole genome sequencing was performed on the stock virus as well as virus present in the supernatant in cell culture infections.** For whole genome sequencing of the virus, purified RNA was subject to cDNA synthesis using a Maxima H minus ds cDNA kit (ThermoFisher Scientific, USA) as per manufacturer instructions; purified using Agencourt Ampure XP magnetic beads (Beckman Coulter Canada, LP), re-suspended in sterile water and quantified fluorometrically via PicoGreen prior to library generation with Nextera XT DNA Library Preparation Kit and Index kits (Illumina Inc., USA). Libraries were quantified following fragmentation and indexing with the Agilent 2200 Tapestation (Agilent Technologies, USA) following manufacturer instructions. Libraries for each sample were normalized to 2 nM and pooled for sequencing with 1% phiX and 1% in-house control spikes. Final concentrations of library pools for sequencing was 20 pM. The Illumina 300-cycle reagent kits with V2 chemistry was used on the MiSeq Illumina platform to generate paired-end sequence data. Adapters and low-quality sequences were removed with Trimmomatic [24]. The program snippy v3.2-dev (https://github.com/tseemann/snippy) was used to align the trimmed reads to the reference CCHFV genome, to estimate coverage and to identify single nucleotide polymorphisms (SNPs), requiring a minimum base quality of 20, minimum depth of coverage of 10 and minimum alternate allele fraction of 0.9 to call a SNP. Alignments were manually inspected for potential SNPs in regions with less than 10 fold depth of coverage.

### Real-time PCR for mRNA validation

Select mRNAs were validated using TaqMan Gene Expression Assays (ThermoFisher Scientific). Total RNA (50 ng) was reverse transcribed using the High Capacity cDNA Reverse Transcription kit (ThermoFisher Scientific) and 2 uL of cDNA reaction was used in the real-time PCR reaction containing TaqMan Universal PCR Master Mix II, No AmpErase UNG (ThermoFisher Scientific) following manufacturer’s recommendations. Data was normalized to the C_T_ values of GAPDH and analyzed using the 2-ΔΔC_T_ method. Data is represented as mean ± standard deviation from 3 biological replicates.

## Results

### RNA-Seq can identify host transcriptional changes in CCHFV-infected cells

In order to simultaneously capture both host response and potential viral evolution, cells were infected with CCHFV at a MOI of 0.1. RNA was isolated at 1- and 3-days post-infection (DPI) from both cell lines. In parallel, RNA was also extracted from uninfected cells. Illumina-based deep sequencing generated a total of 976 million reads from all samples, including 496 million from HepG2 cells (241 million from infected, 255 million from uninfected) and 480 million from Huh7 cells (238 million from infected, 242 million from uninfected). An average of 97.9% of reads in each library mapped to the human genome (range 96.9–98.7%). The host gene expression profile in both liver cell lines was determined using DESeq2 [28] and genes with a greater than 1.5-fold change in expression are presented. The gene expression profiles differed throughout the time-course of infection, with only modest changes observed in both cell lines at 1 DPI, with more notable dysregulation by 3 DPI, (Table 1). Verification of select gene expression was also performed using qPCR (S1 Fig). Additionally, infection resulted in greater upregulation of genes than downregulation when compared to uninfected controls. The subset of genes that increased in abundance by ≥ 1.5-fold coincident with infection was the focus of further analysis (Table 2), which demonstrates specific changes in pathways in both HepG2 and Huh7 cell lines when infected with CCHFV.

**Table 1:** Number of upregulated and downregulated genes at day 1 and 3 post-infection (DPI).

Cell Lines	1 DPI		3 DPI	
	Upregulated	Downregulated	Upregulated	Downregulated
HepG2	12	0	34	3
Huh7	10	3	177	104

**Table 2:** Kinetic changes in pathway type at 1 and 3 days post-infection by cell line.

Days Post-Infection	Pathway Name	Entities Found (^a^)	
		HepG2	Huh7
1	Immune System	15 (3.4x10^-6^)	10 (9.5 x10^-3^)
	Interferon alpha/beta signalling	15 (1.1x10^-16^)	8 (6.7x10^-10^)
	Interferon Signalling	15 (1.1x10^-16^)	8 (6.5x10^-7^)
	Cytokine Signalling in Immune system	15 (6.0x10^-10^)	8 (2.2x10^-3^)
	Interferon gamma signalling	4 (8.1x10^-4^)	
	Antiviral mechanism by IFN-stimulated genes	3 (4.1x10^-3^)	3 (3.2 x10^-3^)
	ISG15 antiviral mechanism	3 (4.1x10^-3^)	3 (3.2 x10^-3^)
	Negative regulators of DDX58/IFIH1 signalling		2 (2.8 x10^-2^)
3	Immune System	36 (2.7x10^-9^)	83 (3.1x10^-4^)
	Cytokine Signalling in Immune system	33 (1.1x10^-14^)	66 (2.8x10^-9^)
	Interferon Signalling	31 (1.1x10^-16^)	43 (1.8x10^-15^)
	Interferon alpha/beta signalling	23 (2.6x10^-13^)	26 (2.6x10^-13^)
	Interleukin-4 and 13 signalling		21 (1.3x10^-9^)
	Interferon gamma signalling	15 (1.3x10^-12^)	23 (2.1x10^-9^)
	Interleukin-10 signalling		10 (1.5x10^-4^)
	Platelet degranulation		8 (1.1x10^-3^)
	Response to elevated platelet cytosolic Ca^2+^		8 (8.8x10^-3^)
	Regulation of Insulin-like Growth Factor (IGF) transport and uptake by Insulin-like Growth Factor Binding Proteins (IGFBPs)		6 (2.9x10^-2^)
	Post-translational protein phosphorylation		6 (6.1x10^-3^)
	Senescence-Associated Secretory Phenotype (SASP)		5 (3.7x10^-2^)
	Antiviral mechanism by IFN-stimulated genes	4 (1.5x10^-2^)	
	ISG15 antiviral mechanism	4 (1.5x10^-2^)	
	Interleukin-18 signalling		3 (4.5x10^-4^)
	RUNX3 regulates CDKN1A transcription		3 (3.7x10^-2^)
	Regulation of IFNA signalling	2 (3.9x10^-2^)	
	RUNX3 Regulates Immune Response and Cell Migration		2 (1.6x10^-2^)
	TGFBR2 MSI Frameshift Mutants in Cancer		1 (2.4x10^-2^)

^a^p-value

### CCHFV alters multiple host pathways

**Fig 1:**
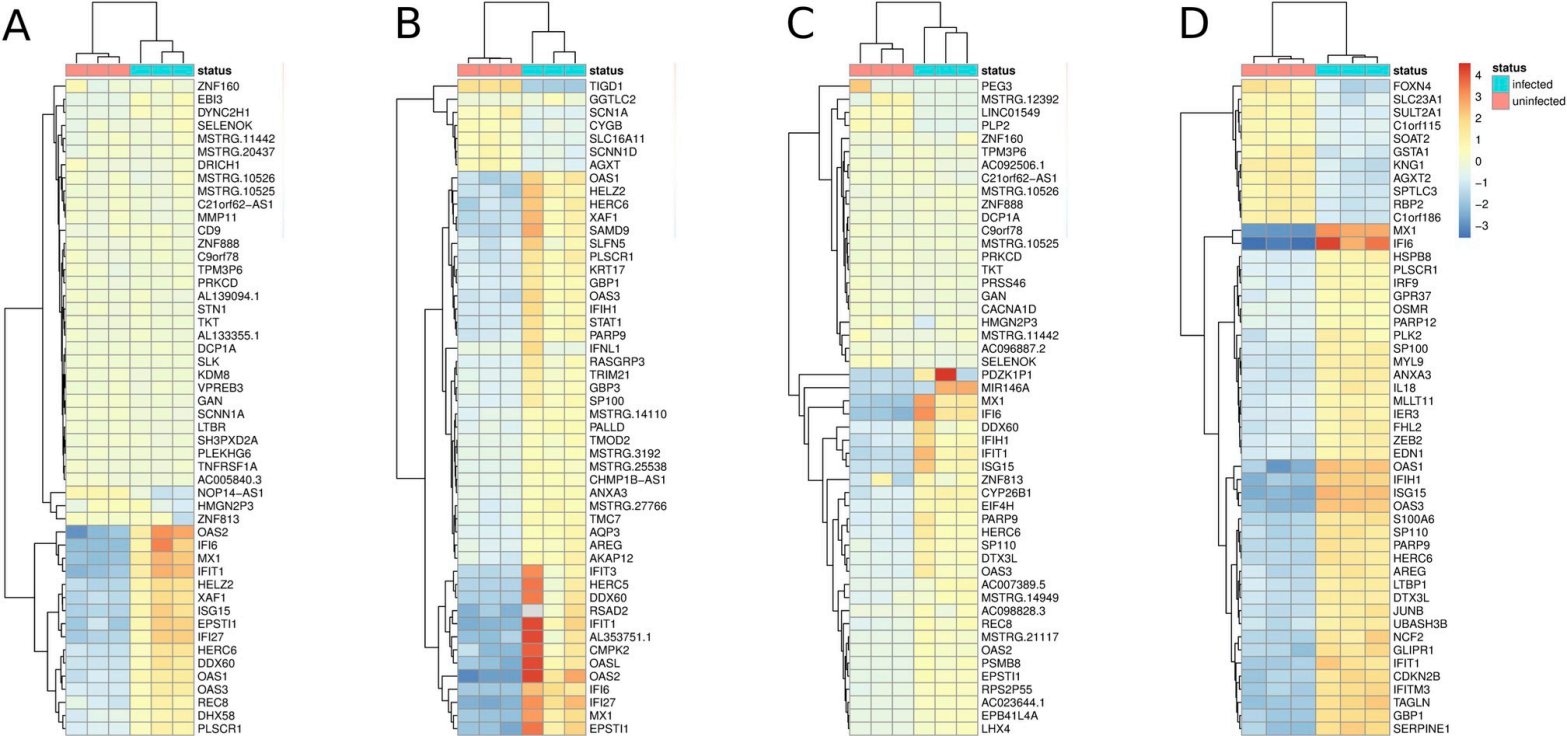
Dysregulation of gene expression 1 and 3 days post CCHFV infection. Comparison of A), B) HepG2 and Huh7 C), D) uninfected (orange square and infected with CCHVF (light blue square) at A), C) 1 and B), D) 3 DPI. Scale represents fold-change from -3 (dark blue square) (3-fold downregulation) to +4 (red square) (4-fold upregulation). Data is from three biological replicates.

We investigated the transcriptional response in HepG2 and Huh7 cells following CCHFV infection with the aim of identifying potential gene expression networks. Gene expression changes were identified using DESeq2 [28]. Biological processes that are perturbed after infection are shown in Fig 1. Overall, expression profiles suggest an induction of the antiviral response in hepatocytes.

**Fig 2:**
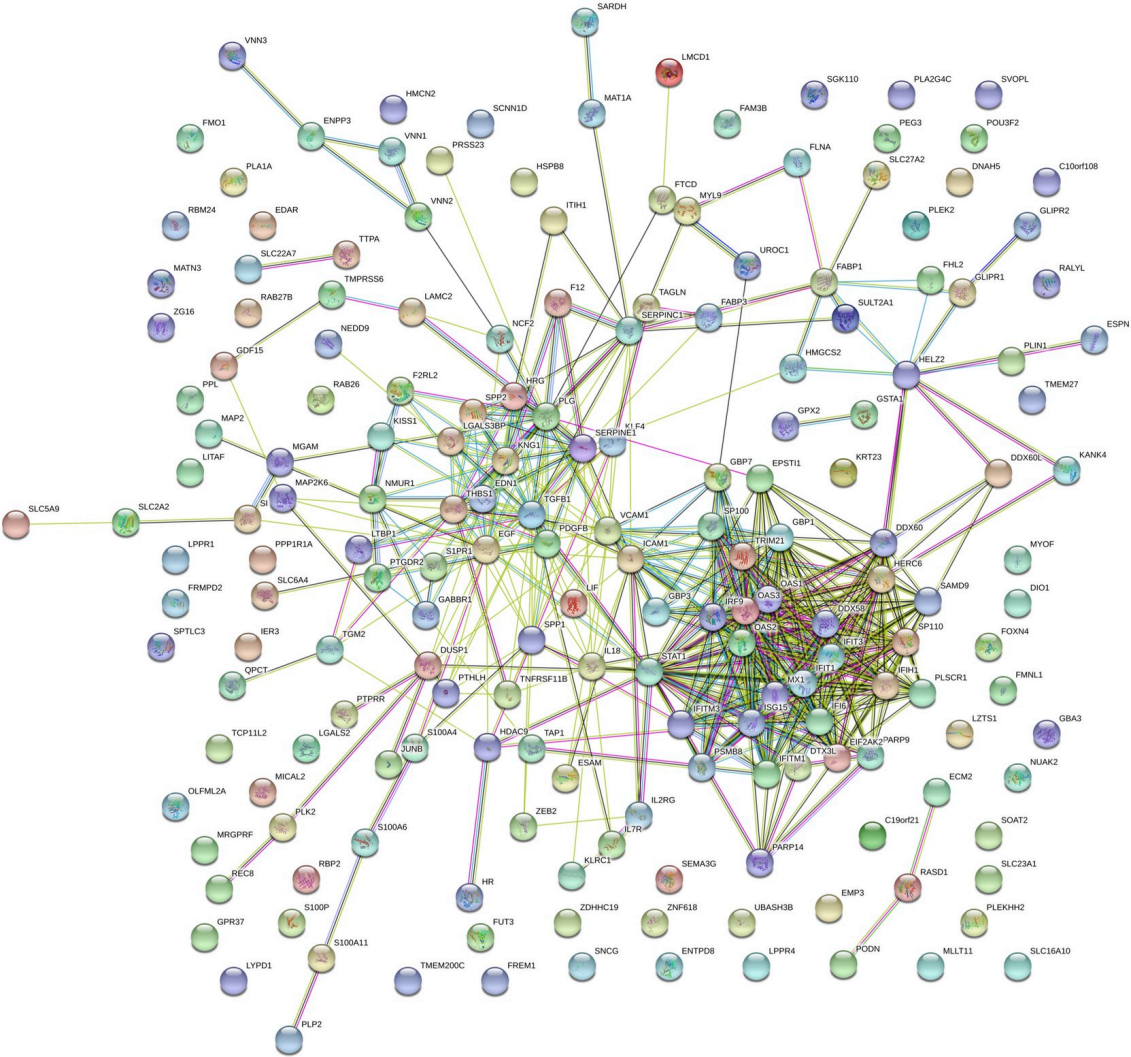
Networks of interacting genes identified using STRING from the RNA-seq results from Huh7 cells at 3 DPI.

*In silico* analysis, using Reactome Pathway Database (www.reactome.org) identified differentially expressed genes that are enriched in particular cellular pathways following viral infection (Fig 2). The pathways that were identified at one and three DPI are shown in Table 2. Interestingly, gene expression profiles were similar in both cell lines, and had common pathways at both time points. Notably, the interferon signalling pathway was one of the top pathways identified for both cell lines at both time points, confirming the importance of the interferon response to CCHFV. Additionally, the induction of interleukin and cytokine pathways is suggestive of a pro-inflammatory response; similar to what has been reported for the infection of human monocytes with Rift Valley Fever virus, a related bunyavirus [31].

Two pathways involved in platelet function were also upregulated in Huh7 cells, one of which is associated with platelet degranulation. This is interesting related to the role of platelets in viral hemorrhagic fever pathogenesis, as degranulation has been reported in non-human primates infected with Ebola virus [32]. Moreover, bunyaviruses have been shown to directly bind platelets [33, 34].

### CCHFV induces antiviral response genes in liver cells

Previous groups have shown that CCHFV is a potent inducer of the interferon response, and the RIG-I pathway [35]. A number of genes that have been identified by other groups were also observed to be upregulated in our study [35]. Notably, the gene encoding MX1, which has been shown to modulate viral replication in Vero cells [36], was upregulated in all cell lines at all time points; *IFIT1* and *IFI6* and were also all upregulated at 1 DPI Table 3. At 3 DPI, numerous interferon-stimulated genes were also dysregulated. Notably *DDX60*, which encodes a cytosolic component of the RIG-I signalling pathway, was significantly induced in both cell lines, and this has pathway been associated with the host response to CCHFV [35]. Furthermore, *OAS2*, which is associated with the antiviral response to RNA was also upregulated. Interestingly, *PTPRR* was induced in Huh7 cells. This has been associated with the TLR9 signalling cascade, and polymorphisms in *TLR9* gene have been associated with poor outcomes in patients [37]. Overall, the expression profile in both cell lines following infection indicates CCHFV induces multiple genes and networks associated with the antiviral and interferon response.

**Table 3:** Top upregulated genes at 1 and 3 days post-infection by cell line.

Days Post-Infection	Gene	Function	Fold-Change	
			HepG2	Huh7
1	*DDX60*	RIG-I signalling pathway	9.2	
	*EPSTI1*	Epithelial stromal interaction 1, plasminogen regulation	6.7	
	*OAS2*	ds-RNA antiviral response	6.2	
	*IFI6*	Interferon alpha inducible protein, regulation of apoptosis	4.8	5.1
	*IFI27*	Interferon alpha/beta signalling	4.8	
	*IFIT1*	Regulator of Type I interferon production/Type I interferon-signalling pathway	4.5	3.2
	*MX1*	Myxovirus (influenza virus) resistance 1, interferon-inducible protein	4.5	4.0
	*XAF1*	Type I interferon signalling pathway	3.6	
	*HELZ2*	Cellular lipid metabolic processes	3.5	
	*HERC6*	Protein ubiquitination		3.2
	*IFIH1*	Regulator of type I interferon production		3.0
	*ISG15*	Type I interferon signalling pathway	3.2	2.8
	*PARP9*	Type I interferon signalling pathway		1.8
3	*GGTLC2*	Glutathione metabolic process	39.7	
	*IFNL1*	Cytokine mediated signalling	28.8	
	*DDX60*	RIG-I signalling pathway	11.2	6.8
	*HERC5*	cytokine mediated signalling, ubiquitination	11.0	
	*RSAD2*	Type I interferon signalling pathway	9.6	
	*OASL*	Type I interferon signalling pathway	8.7	
	*EPSTI1*	Epithelial stromal interaction 1, plasminogen regulation	8.2	8.6
	*RASGRP3*	Ras protein signal transduction	7.8	
	*CMPK2*	pyrimidine nucleotide metabolism	7.5	
	*OAS2*	ds-RNA antiviral response	7.3	9.4
	*IFI6*	Interferon alpha inducible protein, regulation of apoptosis		8.3
	*IL2RG*	Response to IL-2 signalling		7.9
	*CER1*	Bone morphogenic protein signalling pathway		7.6
	*PTPRR*	TLR signalling pathways (TLR2,5,9,10)		7.6
	*GBP3*	Interferon signalling pathway, apoptosis		7.4
	*ASB2*	Protein ubiquitination		6.8
	*CLIP2*	CAP-GLY domain containing linker protein 2, regulator of wound healing		6.2

### CCHFV targets genes important for platelet regulation

**Fig 3:**
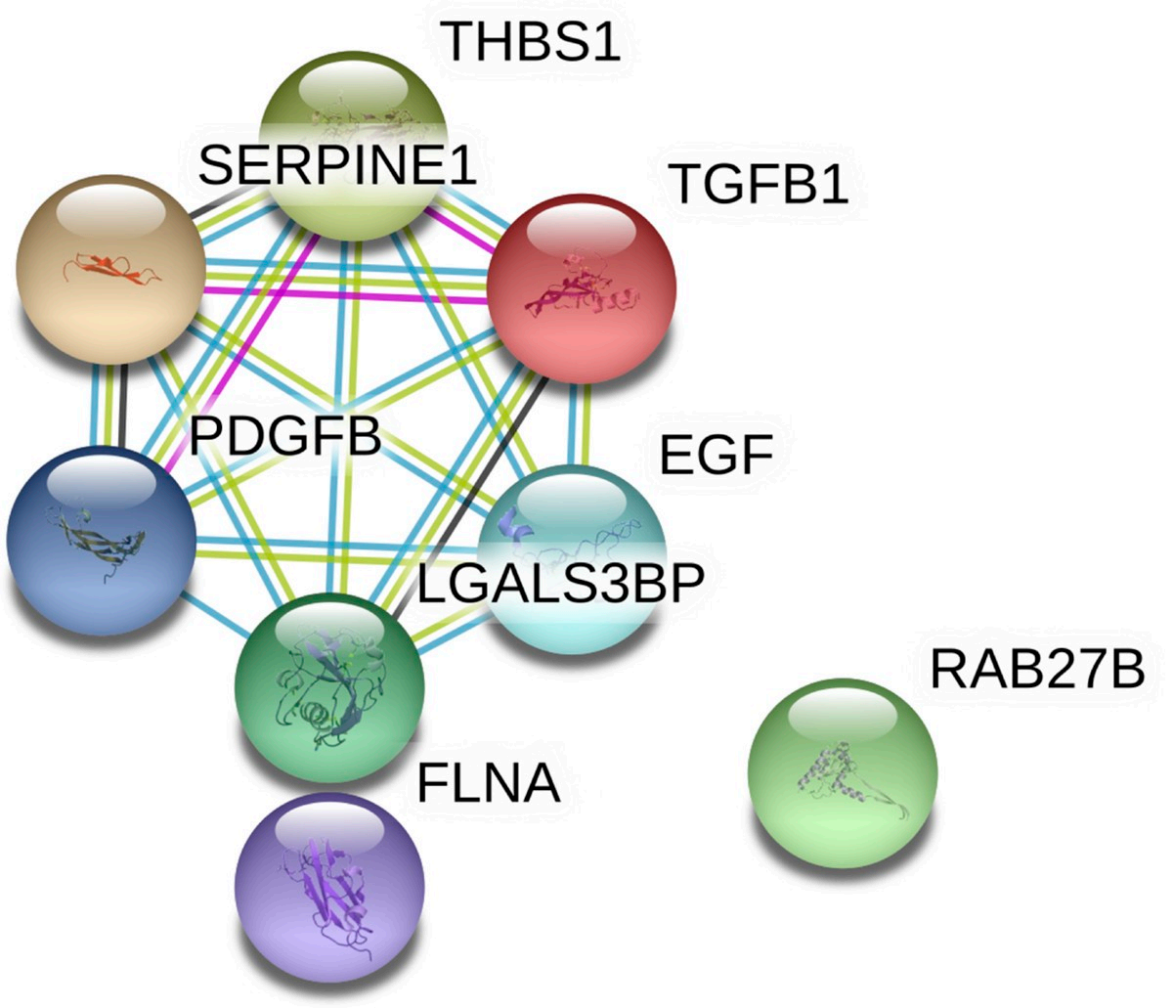
Networks of interacting genes involved in platelet degranulation and activation pathways identified using STRING from the RNA-seq results from Huh7 cells at 3 DPI.

Thrombocytopenia is a hematological hallmark of CCHFV disease, and platelet counts have been reported to be significantly lower in CCHFV patients, compared to healthy controls [38]. The liver plays an important role in platelet regulation, and as noted above, several pathways that were dysregulated are associated with platelet function (Table 3 and Fig 3).

The genes encoding several cytokines that are involved in abnormal clot formation and platelet activation were upregulated. These included *CXCL8* and *EGF*, both of which further activate platelets. Additionally, *CXCL8* has been demonstrated by others to be upregulated following infection [7]. *EDN1* showed an increase in expression. This gene encodes endothelin-1, which has been shown to have elevated expression in children with CCHFV, and negatively correlates with platelet counts [39]. Interestingly, this study also reported increased serum levels of IL-2 receptor, and our data showed 7.9-fold increase in *IL2RG* in Huh7 cells.

Upregulation of *ICAM* was also noted. The soluble form of this protein has been suggested to be a marker of poor outcome potentially due to its association with vascular damage [40]. Furthermore, there was a 4-fold increase in *SERPINE1*, a gene which encodes tissue plasminogen activator inhibitor-1 (PAI-1). Increases in PAI-1 have been shown to correlate with disease severity for both CCHFV and Hantavirus cardiopulmonary syndrome [41, 42]. This could potentially be related to the increase in small clot formation leading to disseminated intravascular coagulation, a hallmark of CCHFV severity [42]. Additionally, the interferon gamma signalling pathway was a key activated pathway. IFN-γ is pro-inflammatory and may add to thrombocytopenia.

### Viral sequencing of CCHFV by RNA-Seq

**Fig 4:**
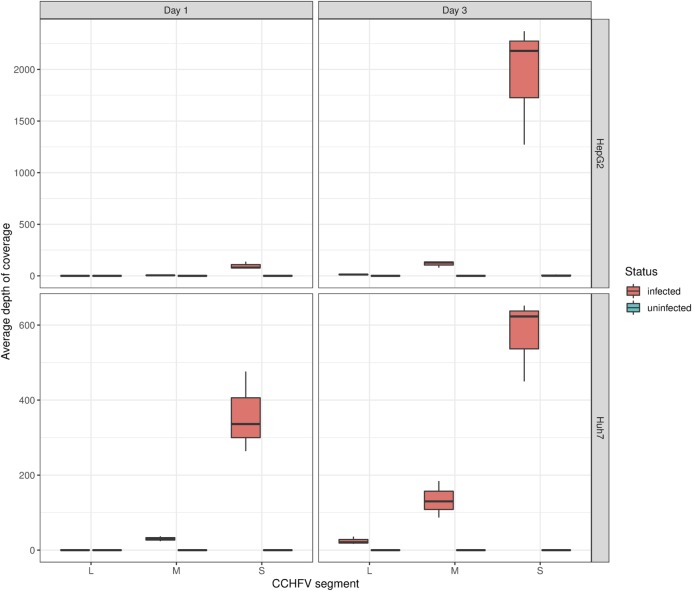
Reads aligned to the CCHFV genome in infected Huh7 and HepG2 cells at 1 and 3 days post-infection.

The ability to examine both the host transcriptome and the pathogen from the same sample allows for a better understanding of infection-specific profiles [14]. Moreover, from a diagnostic perspective, it allows for the rapid identification and genotyping of the virus. Viral replication in both cell lines was confirmed by qPCR, and viral reads were isolated from both infected cell lines at multiple time points (S2 Fig). A total of 11,396 (Huh7, 1 DPI) and 28,238 (Huh7, 3 DPI), compared to 2928 (HepG2, 1 DPI) and 55,424 (HepG2, 3 DPI), reads were aligned to the CCHFV genome. Coverage of the three viral segments (L, M, and S), reported as the average depth of coverage per base averaged across triplicates, varied markedly within infected cells, (Fig 4). At 3 DPI, the S segment, encoding the abundant nucleocapsid protein, had the highest average coverage in both cell lines at 1940x (HepG2) and 575x (Huh7) followed by the M segment, encoding viral envelope glycoproteins with an average of 116x (HepG2) and 134x (Huh7) coverage. The L segment, encoding the viral RNA polymerase, had the lowest coverage at 13x (HepG2) and 25x (Huh7). Analysis of these reads correctly identified the virus as being the IbAr10200 strain. Additionally, no mutations were identified during the course of infection. This was confirmed by deep sequencing specific for the virus performed in parallel, which showed viral reads remained identical to stock virus used for infection. Compared to RNA-Seq, pathogen specific sequencing from both infected cell lines at three timepoints achieved greater average coverage of the S, M and L segments, with an average coverage of 800X with significant intra-sample variability (L segment mean 896, range 19–3504; M segment mean 800, range 5–3002; S segment mean 826, range 11–2992). In summary, this demonstrates that RNA-seq can identify the strain of CCHFV in host samples, which can have implications for diagnostics and clinical epidemiology.

However, applications, which require uniformly high depth of coverage for all segments, such as high resolution phylogenomic studies during outbreaks, may benefit from additional pathogen specific sequencing.

## Discussion

In this study we used dual RNA-seq to simultaneously sequence CCHFV and identify host genes that are dysregulated following infection. Through a combination of bioinformatics and gene profiling we were able to identify host pathways associated with CCHFV pathogenesis in liver cell lines. While several groups have identified genes with altered expression in CCHFV infection [43–45], to our knowledge this is the first time next-generation sequencing has been applied to examine the host-response *in vitro* to CCHFV. Our data indicates that CCHFV infection of hepatocyte cell lines induced robust antiviral and pro-inflammatory responses. However, it has been demonstrated that induction of certain antiviral factors does not restrict the virus. For example, *MX1*, which was upregulated in both of our cell lines, was shown by Andersson *et al*. to decrease CCHFV replication *in vitro*, but not completely prevent it [36]. Other groups have also shown viral replication despite the induction of *ISG15* and *IFNβ1* [35], suggesting the virus likely overcomes these pathways. Recent findings from Scholte *et al*. have demonstrated that the CCHFV encodes an ovarian-tumor superfamily (OTU) protease that has dual deubiquitinase and deISGylase activity (mediated by the L-protein), which is used by the virus to suppress the innate immune response [46]. This suggests that although CCHFV induces a robust antiviral response in liver cells, the virus may have a mechanism to moderate this response. Moreover, all of the cellular targets of the OTU protease have not been identified, and the induced genes in our data set provide potential targets for further investigation. Additionally, a strong pro-inflammatory response may contribute to the endothelial cell and vascular leakage, and this is supported by clinical observations that high-levels of pro-inflammatory cytokines, such as CXCL8, which had elevated mRNA expression in our experiment, are seen in fatal cases [47]. A limitation of our study was the use of only established cell lines, and not primary hepatocyte cultures. Although HepG2 and Huh7 cells both express a similar repertoire of TLR genes, expression levels differ for between cell lines, and TLR8 is not expressed in either cell line [48]. Furthermore, *IFN-β* expression in response to viral infection is known to be absent in Huh-7 cells due to defects in the TLR3 and RIG-I pathways [49], and this likely accounts for the greater number of genes associated with the RIG-I pathway induced in HepG2 cells in our experiments. While primary hepatocytes are difficult to culture, studies will provide a greater understanding into the role of different host pathways in CCHFV infection.

The induction of genes associated with platelet regulation provides further insight into the characteristic bleeding and thrombocytopenia that is associated with CCHFV infection. Platelets maintain the integrity of the vascular system, and have been shown to be critical for the pathogenesis of viral hemorrhagic fevers [33]. *CXCL8*, *ICAM1* and *EDN1*, all of which were induced in our experiments, are associated with vascular leakage and with worse outcomes in patients [40, 47, 50]. Excessive release of cytokines and soluble markers likely dysregulate platelet activation. Moreover, the induction of pro-inflammatory and interferon pathways could also contribute to the vascular damage. Interestingly, increased levels of ICAM1 protein were also reported by Fraisier and colleagues in CCHFV infection of HepG2 cells, and hypothesized to play a role in the oxidative stress response to the virus [51]. A limitation of our study is the use of the prototypical IbAr 10200 strain, which has undergone significant passaging and lab adaptation and is likely more attenuated than currently circulating strains [9, 52]. Although use of this strain permits comparison to other laboratory findings (due to it’s widespread use in Western labs), further studies to compare transcriptomic profiles from CCHFV strains associated with low mortality, such as AP92, to more virulent isolates, performed in both cell lines and primary cell cultures are needed to provide additional insight into viral pathogenesis [53–55].

From a diagnostic perspective, RNA-Seq offers considerable promise, especially for CCHFV, and other viral hemorrhagic fevers. The ability of RNA-Seq to recover to provide high coverage of the M segment of CCHFV, which is the segment used for virus characterization [56], would allow a single test to provide virus typing at the same time as characterizing the host response; and potentially disease progression. A recent study has identified *HGMB1* as a biomarker that is dysregulated in numerous viral hemorrhagic fevers, including CCHFV [57]. Thus, although viral load has been identified as a strong predictor of patient outcome, other clinical markers, such as low platelet count, has also been associated with poor outcome [11]. Thus, it is conceivable that that a gene expression signature associated with a clinical marker, could be determined; perhaps even before biochemical indicators, and could predict patient prognosis. Considering, the high-consequence nature and cost of animal experiments to study CCHFV, dual RNA-seq is a method that can provide insight into pathogenesis using clinical samples; thereby increasing our understanding of the disease process. Our study serves as proof-of-principal that next-generation mRNA sequencing is a method to evaluate the interplay between the host and virus. Furthermore, sequencing the virus over time may also lead to the identification of novel strains or variants, especially of clinical importance in outbreak settings.

## Supporting information

S1 Fig Transcription of genes was analyzed using quantitative RT-PCR validation (qPCR) with GAPDH as a housekeeping gene and expressed as a fold change compared to uninfected cells using 2-ΔΔCt method. Data shown represents the mean of three biological replicates, and error bars represent the standard deviation.
(TIF)

S2 Fig Viral replication in Huh-7 and HepG2 cell lines. Cells were infected at an MOI = 0.1 and at various time points genome equivalents were determined by RT-PCR Experiments were performed in triplicate and error bars represent the standard deviation.
(TIFF)
