# Supplementary figures and images for: Dual RNA-Seq characterization of host and pathogen gene expression in liver cells infected with Crimean-Congo Hemorrhagic Fever Virus
Source: PLoS Negl Trop Dis. 2020 Apr 6;14(4):e0008105. doi: 10.1371/journal.pntd.0008105 (PMC7162549; doi:10.1371/journal.pntd.0008105)

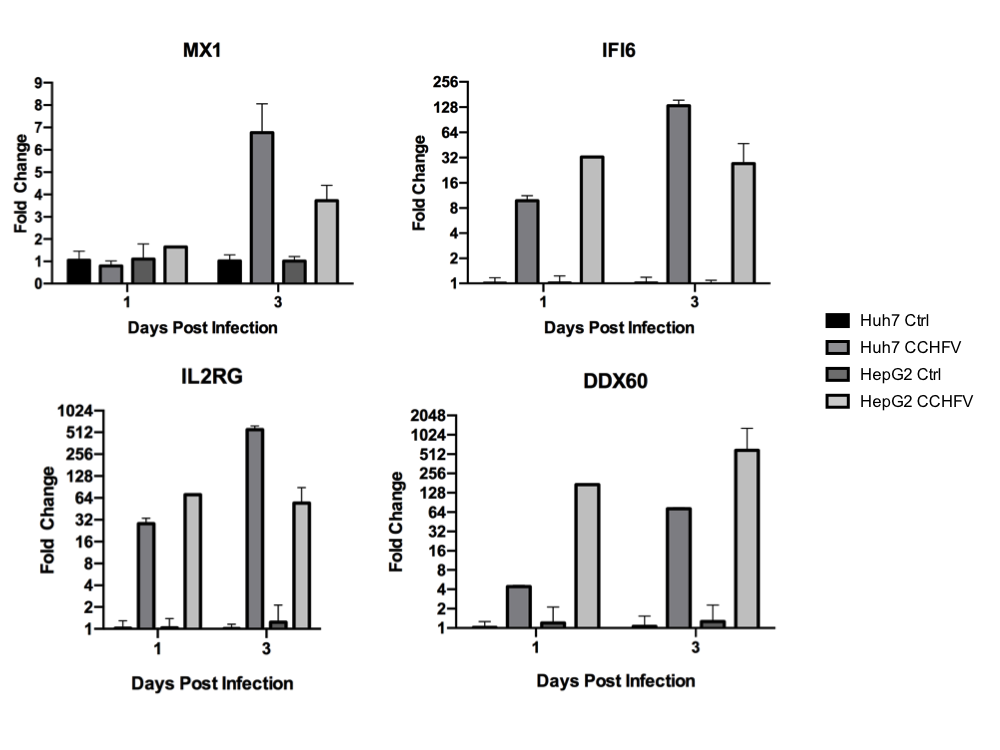

Supplement: S1 Fig — Data shown represents the mean of three biological replicates, and error bars represent the standard deviation. (TIF) [file pntd.0008105.s001.tif]

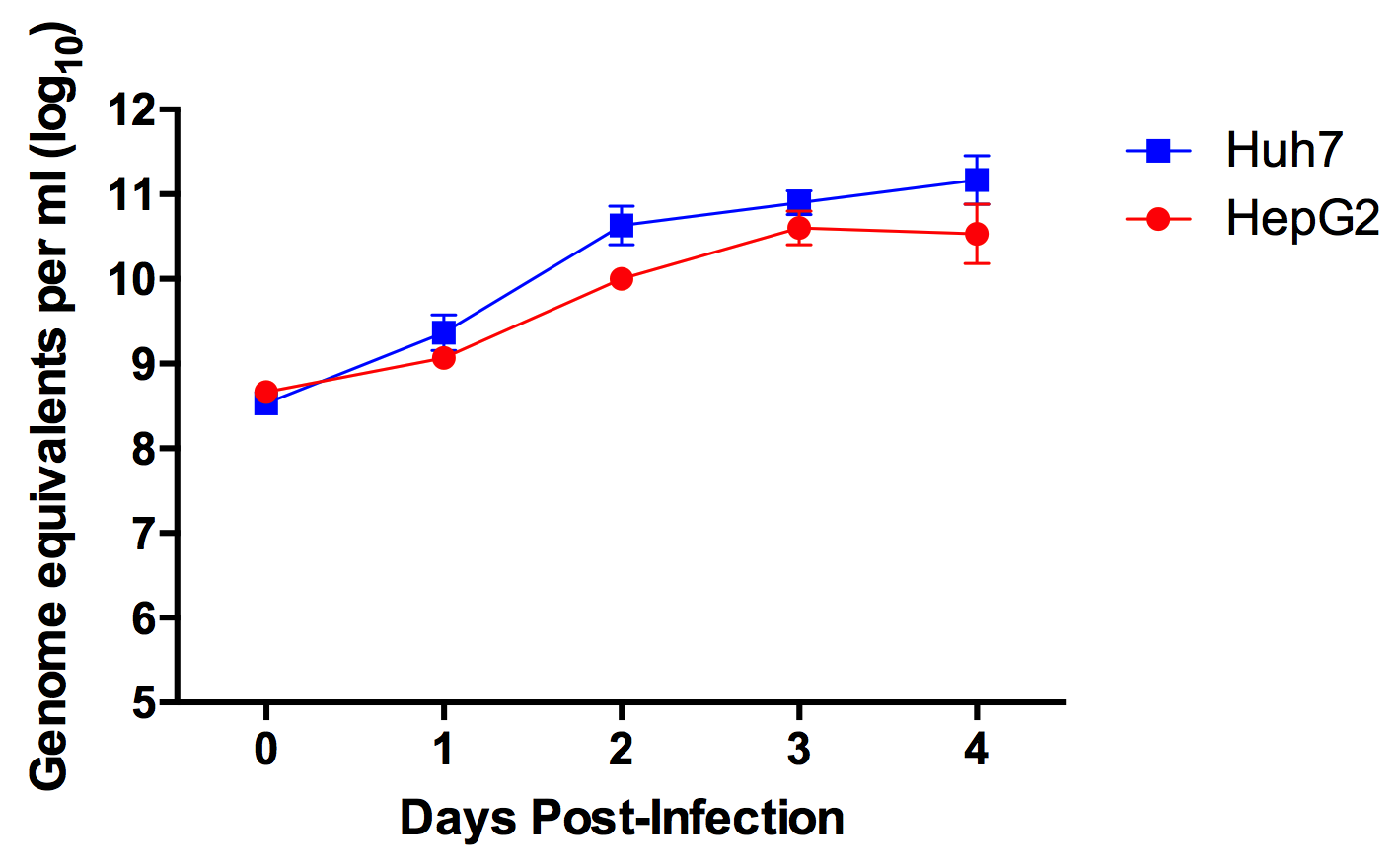

Supplement: S2 Fig — Cells were infected at an MOI = 0.1 and at various time points genome equivalents were determined by RT-PCR Experiments were performed in triplicate and error bars represent the standard deviation. (TIFF) [file pntd.0008105.s002.tiff]
